# The Werther effect of celebrity suicides: Evidence from South Korea

**DOI:** 10.1371/journal.pone.0249896

**Published:** 2021-04-28

**Authors:** Jeongmin Ha, Hee-Seung Yang

**Affiliations:** School of Economics, Yonsei University, Seoul, Republic of Korea; Sogang University (South Korea), REPUBLIC OF KOREA

## Abstract

Since 2003 Korea has experienced the highest suicide rate among OECD countries. One of the societal risk factors that triggers suicide is the contagious nature of suicide. This paper empirically examines the effect of celebrity suicide reports on subsequent copycat suicides, using daily suicide data and information of highly publicized suicide stories in Korea from 2005 to 2018. The findings from the Poisson regression model suggest that the number of public suicides soars after media reports on celebrity suicides. On average, the number of suicides in the population increased by 16.4% within just one day after the reports. Further analysis reveals that female and younger subgroups are more likely to be affected by celebrity suicides. Moreover, the public reacts more strongly to suicide incidents of celebrities of the same gender and even imitates the methods of suicide used by celebrities. This paper highlights the significance of careful and responsible media coverage of suicide stories to prevent copycat suicide. For policymakers, it is crucial to implement regulations not only for traditional media but also for new media where younger people can freely access unfiltered information.

## Introduction

**Fig 1:**
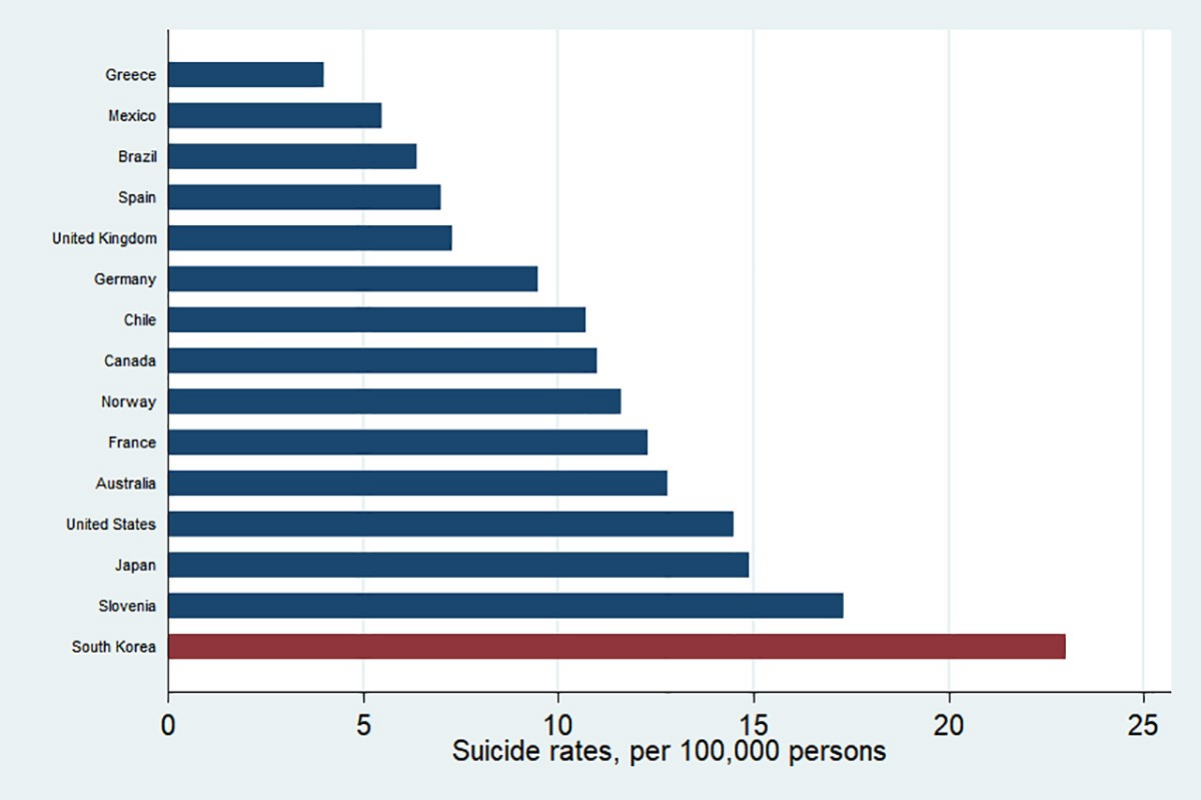
Suicide rates, per 100,000 persons, 2018 or latest available. Note: Suicide rates are defined as the deaths deliberately initiated and performed by a person in the full knowledge or expectation of its fatal outcome. Source: OECD (2020), Suicide rates (indicator). doi: 10.1787/a82f3459-en (Accessed on 06 November 2020).

Globally, suicide rates among youth have increased significantly over the past several decades [1, 2]. Among others South Korea (hereafter Korea) has the highest suicide rate as shown in Fig 1: as of 2018, 23 out of 100,000 Koreans committed suicide and the number is the highest among OECD member countries whose average suicide rate was 11.2. Suicide is now one of the leading causes of death among young adults in Korea [3]. In particular, the contagious nature of suicide is one of the societal risk factors that triggers suicide, especially for adolescents [4–8], although it is controversial that negative emotion or risky behavior are contagious [9–11].

Research on suicide has important policy implications because suicide is a serious social problem that incurs significant psychological and financial costs for bereaved family members and their society [12]. The study of demographic, social, and economic determinants of suicide dates back to Durkheim [13]. Durkheim’s claim of suicide imitation or suicide suggestion has been increasingly examined by various fields of researchers (for example, see [14]). However, the question on suicide imitation has not been studied rigorously in the Korean context. Time series observation in Korea shows that the number of public suicides significantly increased after the two most publicized suicide incidents of two celebrities, Choi and Roh, who passed away on October 2008 and May 2009 [15, 16]. Suicides of celebrities often cause a surge in suicide rates among people of the same gender and age group, and many of them even use the same method of suicide as the celebrities did [17]. This paper studies whether there are indications of the Werther effect, which refers to imitative suicidal behavior in Korea. The term “Werther effect” was first used by Phillips to show imitation of celebrity suicide, i.e., whether the number of suicides increased after a suicide story was publicized. Phillips finds a nationwide increase in suicides in the U.S. and in Britain after publicized suicides, which is contrary to Durkheim’s claim of the irrelevance of timing on suicide [13, 18].

Since the first empirical research on the Werther effect was conducted by Phillips, the claim has been reinforced in the U.S. that highly-publicized suicide stories have a negative impact on the public [18–22]. For example, Stack finds that suicide stories of entertainers and politicians led to imitative suicides using U.S. data of suicide between 1948 and 1983 [21]. A continuous empirical analysis of the Werther effect has been performed in other countries as well. Cheng et al. investigate the case of Taiwan, suggesting that reporting a suicide of a Taiwanese celebrity caused public copycat suicides, especially for young males using the same method (hanging) [23]. Ladwig et al. and Hegerl et al. find evidence that the number of railway suicidal acts in Germany increased significantly after the railway suicide of a certain celebrity [24, 25]. According to Ueda et al., the general public in Japan is most affected by the suicides of nationally recognized politicians, followed by entertainers [26]. In more recent studies, Ueda et al. show that copycat suicides increase in Japan only when suicides of celebrities elicit a large reaction from Twitter users [27]. Furthermore, Bridge et al. find that the release of a Netflix series, which deals with the story of an adolescent girl who commits suicide, was associated with an increase in suicide rates among teenagers in the U.S. [28]. In the Korean context, Fu and Chan suggest that the magnitude of the impact of celebrity suicides on the public was positively associated with the amount of media coverage using weekly suicide counts [29]. Kim et al. also find evidence of the Werther effect in Korea by focusing on the suicides of two specific celebrities (an actress and the former President who committed suicide in October 2008 and May 2009) [16].

Nevertheless, existing studies of the Werther effect have several limitations. First, studies using monthly or weekly data cannot exactly distinguish whether public suicides were caused by celebrity suicides or by other events. To cope with this problem, we employ daily suicide data. Jonas also uses daily suicide counts between 1968 and 1980 to test the Werther effect in Germany, and thus our paper extends the prior effort by investigating the new media environment in the 2000s [30]. In particular, the rapid development of mass media and Internet technology has increased the amount and speed of information dissemination, and the media now provides a new interactive and communication platform for youth and young adults. Second, a more general analysis is needed because previous studies have focused on investigating the impact of specific celebrity suicides [15–17, 24, 25, 29, 31]. We employ a more comprehensive list of celebrities over a longer period than previous studies. As opposed to previous research, we also consider macroeconomic factors which might affect suicidal behavior such as stock price index, policy changes, celebrity deaths by accidents or illness, and other factors such as weather. Including these variables leads us to distinguish whether the change in the public suicide rate is due to celebrity suicide reports or other circumstances. Finally, we evaluate the impact of the media policy on suicide rates. This analysis provides discussion on how the government could prevent and reduce copycat suicides and suggests policy implications regarding the media regulation. This study will shed light on the importance of media policy because celebrities’ behaviors frequently provoke public feelings through media exposure [32].

## Materials and methods

Daily suicide data were extracted from mortality microdata published by the Korean National Statistics Office. It includes information from 2005 to 2018 for the date of death, gender, age at death, and methods of suicide. In our data, the variable for suicide methods is based on the International Classification of Disease (ICD). The ICD classifies unique codes for causes of death. The codes X60 to X80 in the ICD correspond to suicide methods, and we reclassified them into four types: self-poisoning, hanging, jumping or using deadly objects, and others. The total number of suicides in our sample is 189,985 and the total period we cover is 5,113 days. Summary statistics for the number of daily suicides by age group, gender and method of suicide are given in Table 1.

**Table 1:** Summary statistics for daily suicides.

Variable	Observation (days)	Mean (persons)	Std. Dev.	Min (persons)	Max (persons)
Total	5,113	37.157	9.735	9	87
Ages of 10–29	5,113	4.544	2.572	0	20
Ages of 30–49	5,113	12.918	4.589	1	43
Ages of 50–69	5,113	11.935	4.209	0	34
Ages of 70 or above	5,113	7.758	3.334	0	25
Male	5,113	25.333	7.197	5	56
Female	5,113	11.824	4.494	1	41
Unspecified means	5,113	.404	.704	0	5
Self-poisoning	5,113	10.38	3.993	0	28
Hanging	5,113	18.913	6.665	3	63
Drowning	5,113	1.273	1.208	0	8
Fire, smoke, explosive materials	5,113	.268	.525	0	4
Jumping, deadly objects	5,113	5.918	2.651	0	19

We gathered information about 13 highly publicized celebrity suicides between 2005 and 2018. The celebrities are composed of 11 entertainers and two politicians. In addition to the list, we also present the number of news reported in the biggest search engine in Korea, NAVER, within one month after the celebrity’s death using keywords such as “[celebrity name] death or suicide.” NAVER (http://www.naver.com) is a web portal that has the largest market share in Korea. Even though more celebrities committed suicides during our study period, we selected 13 celebrities whose suicides were reported more than 300 by newspapers on the search engine, in a similar way to Hong and Lee [31]. Hong and Lee chose 11 celebrities whose suicides were cited more than 100 times by major newspapers during a week since the day of the suicide, using the NAVER news search engine [31]. Moreover, we added 10 celebrities (entertainers, politicians, and entrepreneur) on the list who died in ways other than suicide, such as disease or accident. These cases will be used for an analysis distinguishing between the effect of suicide and the effect of accidental death. Table 2 provides the list of celebrities.

**Table 2:** Characteristics of celebrity death incidents: 2005–2018.

Name	Date of suicide	Sex	Age group	Cause of death	# of online news reported (10/14/2020)
Lee, Actress	02/22, 2005	Female	20	Suicide	339
Jeong, Actress	02/10, 2007	Female	20	Suicide	604
Ahn, Actor	09/08, 2008	Male	30	Suicide	3,143
Choi, Actress	10/02, 2008	Female	40	Suicide	4,409
Jang, Actress	03/07, 2009	Female	20	Suicide	3,612
Roh, Politician^a^	05/23, 2009	Male	60	Suicide	2,475 / 26,230
Choi, Actor	03/29, 2010	Male	40	Suicide	1,988
Park, Actor	06/30, 2010	Male	30	Suicide	2,481
Chae, Singer	05/27, 2011	Male	30	Suicide	825
Kim, Actor	06/26, 2016	Male	40	Suicide	586
Kim, Singer	12/18, 2017	Male	20	Suicide	1,356
Cho, Actor	03/09, 2018	Male	50	Suicide	378
Roh, Politician^a^	07/23, 2018	Male	60	Suicide	1,149
Kim, Singer	04/29, 2008	Male	20	Accident	396
Kim, Politician^a^	08/18, 2009	Male	80	Disease	559 / 15,463
Lim, Singer	02/11, 2013	Male	30	Disease	1,356
Yoo, Singer	07/24, 2014	Female	40	Disease	1,472
Kwon & Koh, Singer^b^	09/07, 2014	Female	20	Accident	2,080
Shin, Singer	10/27, 2014	Male	40	Disease	9,505
Kim, Politician^a^	11/22, 2015	Male	80	Disease	1,042 / 14,538
Kim, Actress	04/09, 2017	Female	60	Disease	192
Kim, Actor	10/30, 2017	Male	40	Accident	5024
Koo, Entrepreneur^a^	05/20, 2018	Male	70	Disease	84 / 2,341

^a^ In addition to using the words ‘death’ or ‘suicide’ when searching news, the words ‘Seogeo’ and ‘Byeolse’ were used and the number of online news including these words is also added in the last column. These Korean words are an honorific expression of the death of people who had a high social position such as former president, politician, and head of a conglomerate.

^b^ The number of online news about Kwon, who had been more reported among the two, is listed.

Furthermore, we collected other information from several sources. We use daily weather data provided by the Korea Meteorological Administration which include average temperature, precipitation duration, humidity, and amount of sunshine. We also use daily Composite Stock Price Index from the Bank of Korea, monthly unemployment rates from the Statistics Korea, and quarterly data of real GDP from the Bank of Korea. Finally, monthly divorce rates were retrieved from the Vital Statistics of the Statistics Korea.

Using the data above, we estimate the following Poisson regression model since the dependent variable describes the number of occurrences of daily suicide [33]. It has a setting similar to Ueda et al. [26].

$$\log\left(\mu_{dmy}\right)=\sum_{k=-10}^{10}\beta_{k}R_{dmy,k}+\gamma X_{dmy}+\delta_{w}+\lambda_{y}+\rho_{m},$$ (1)

where *μ*_*dmy*_ is the number of public suicides on day *d* in month *m* of year *y*. *R*_*dmy*,*k*_ is the main independent variable and has the value of 1 if a certain date on day *d* in month *m* of year *y* is *k* days away from a celebrity suicide report, and 0 otherwise. To interpret the magnitude of the effect, the incident rate ratio (IRR) is used, which is expressed as exp(*β*_*k*_). If, for example, IRR on day *k* after the celebrity suicide is 1.257, then the number of suicides on that day increases by 25.7 percent on average [33].

We mainly analyze 10 pre- and post-periods from the celebrity suicide because several studies have shown that the effect lasts about 10 days [22, 26, 27]. However, other studies have provided empirical evidence that the effect could last longer than 10 days for some cases of celebrities [16, 29]. Thus, we also conduct the analysis with wider time windows, 20 and 40 pre- and post-periods.

Existing studies have shown that frequency of suicides may be affected by several risk factors: seasonal variation, calendar year, weather [34, 35], and stock prices [36], unemployment, business cycles and economic growth [37, 38], and divorce rates [39, 40]. Thus, we control for the variables (*X*_*dmy*_) that could affect public suicides such as daily weather conditions, daily stock price index, monthly divorce rates, monthly unemployment rates, and quarterly GDP growth rates.

In addition, the Poisson regression model includes year, month and day-of-week fixed effects and a linear time trend in order to control for impacts of the time factors on suicides. The year fixed effects (*λ*_*y*_) and the month fixed effects (*ρ*_*m*_) are expected to capture influences of any year-specific factors and seasonal variation. The day-of-week fixed effects are also controlled for since a specific day of week could influence the number of suicides. Further, we consider the policy change, “Suicide Reporting Guideline 2.0,” announced on September 9, 2013 [41]. We can observe the effectiveness of the media policy through this variable.

## Results

**Fig 2:**
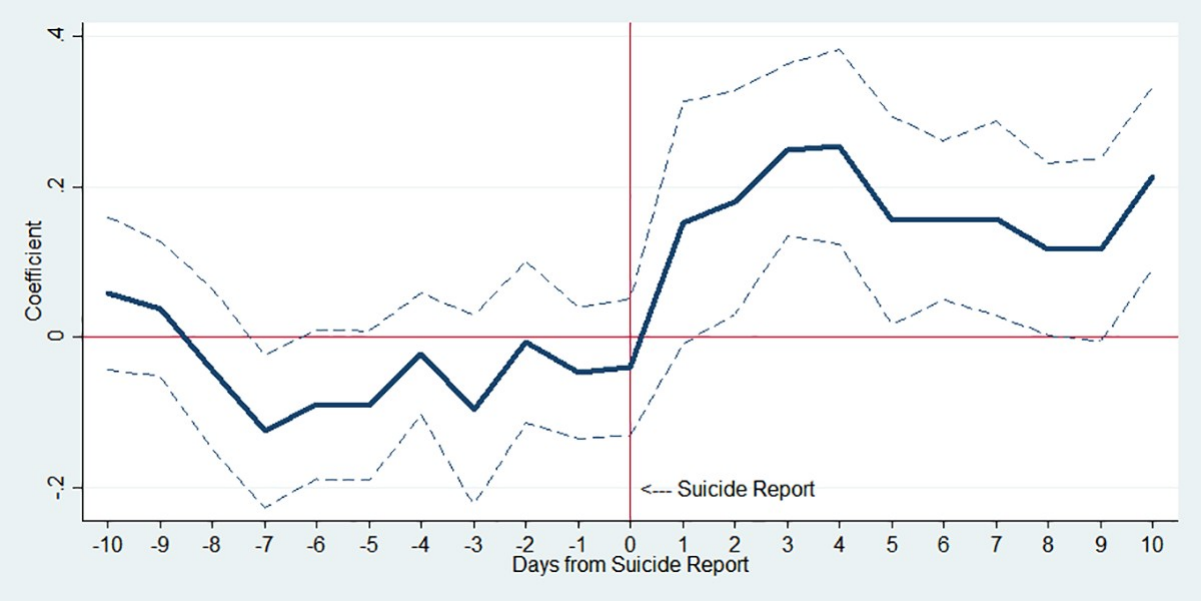
Estimated changes in total suicides before and after media reports on celebrity suicide. Note: The *y*-axis indicates an approximate percent change in public suicide by corresponding day, which is the estimated *β*_*k*_ in Eq (1). The dotted lines indicate 95% confidence interval.

In Fig 2, the estimation results are shown when the whole sample is used. To be specific, the *x*-axis shows the number of days from the news about a celebrity suicide; day 0 refers to the day when a celebrity committed suicide, days 1 to 10 are the post-period, and days -1 to -10 indicate the pre-period. The *y*-axis indicates an approximate percent change in public suicide by corresponding day, which is the estimated *β*_*k*_ in Eq (1). Hence, Fig 2 displays the impact of the 10 pre- and post-periods from the celebrity suicide on imitative suicides.

As expected, there is no notable effect of the celebrity suicide in 10 days before the incident. In the post-period, however, it is shown that highly publicized celebrity suicides are associated with increased public suicides. In column (1) of Table 3, IRR on day 1 is 1.164 (p-value<0.1), implying that on average the number of suicides in the population increased by 16.4% in just one day after the incident. Moreover, IRR on day 4 is the highest at 1.288 (p-value<0.001), and all of IRR on the post-period show a statistically significant increase in the number of suicides.

**Table 3:** Incident rate ratio (IRR) on days away from celebrity death incidents.

	(1)	(2)	(3)	(4)	(5)	(6)
	Overall	Ages of 10 to 29	Male	Female	By Hanging	Not by Suicide
Day 1	1.164*	1.347**	1.130	1.233**	1.539***	1.003
	(0.0956)	(0.186)	(0.0880)	(0.129)	(0.171)	(0.0601)
Day 4	1.288***	1.696***	1.201***	1.466***	1.664***	1.002
	(0.0846)	(0.179)	(0.0854)	(0.158)	(0.146)	(0.0856)
Day 7	1.171**	1.434***	1.126*	1.261**	1.395***	0.971
	(0.0768)	(0.147)	(0.0767)	(0.116)	(0.125)	(0.0647)
Day 10	1.237***	1.487***	1.093	1.532***	1.386***	1.016
	(0.0767)	(0.171)	(0.0796)	(0.0907)	(0.134)	(0.0427)
Average Effect	1.193***	1.342***	1.125***	1.331***	1.429***	0.973
(1–10 days)	(0.0258)	(0.0579)	(0.0251)	(0.0409)	(0.0464)	(0.0196)
Observations	5,113	5,113	5,113	5,113	5,113	5,113

Note: Robust standard errors are in parentheses.

*** p<0.01

** p<0.05

* p<0.1

**Fig 3:**
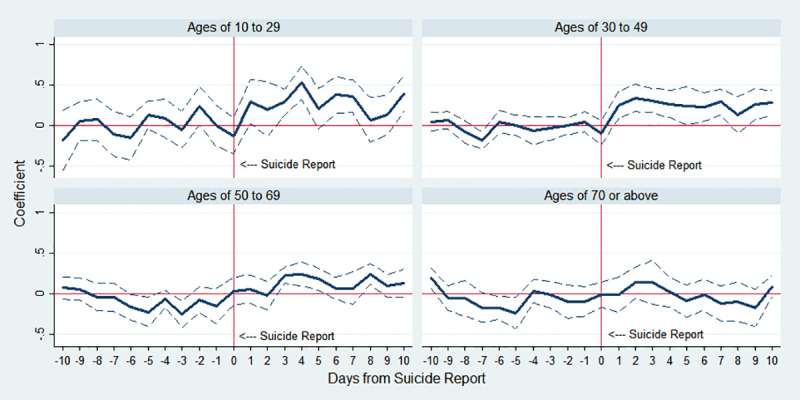
Estimated changes in total suicides by age group before and after celebrity suicide media reports. Note: The *y*-axis indicates an approximate percent change in public suicide by corresponding day, which is the estimated *β*_*k*_ in Eq (1). The dotted lines indicate 95% confidence interval.

Fig 3 provides evidence that the contagious effect of the celebrity suicides is greater for the younger generation than the older generation. In general, the growth of suicide rate has been more substantial for older cohorts in Korea. However, for groups aged more than 50 there are no discernible effects after the publicized suicides, which is consistent with the previous literature [42]. The estimate results for the subsample between the ages of 10 and 29 are given in the top-left panel of Fig 3 and column (2) of Table 3. As the younger subgroup is more likely to be accessible to the media and has interest in celebrities, especially entertainers, the magnitude of the coefficient increases more sharply after celebrity suicides. IRR on day 1 is 1.347 (p-value<0.05), and IRR on day 4 is the highest at 1.696 (p-value<0.001), meaning that the number of suicides in the population increases by 69.6% on average. In the top-right panel of Fig 3, the number of suicides of people between the ages of 30 and 49 also surges immediately after the incidents, but the magnitude is less than that of the younger subgroup; for example, IRR on day 1 is 1.291 (p-value<0.01). Two remaining graphs in the bottom panel of Fig 3 show the estimation results of older subgroups. For the group whose age is between 50 and 69, the coefficient appears statistically significant only a few days after the celebrity suicides, and its magnitude is smaller. Also, the effect does not appear in the oldest group over 70 years of age.

We also conduct a further analysis with wider time windows of 20 and 40 pre- and post-periods, since a few existing studies have shown that the effect could last longer than 10 days for some cases of celebrities [16, 29]. These results are shown in Figs A1 and A2 in S1 Appendix. We are able to confirm that the effects are still statistically significant even after 10 days, but gradually decrease over time. Specifically, Table 4 shows the average effect of celebrity suicides on total suicides over 10, 20, and 40-day post-reporting periods. Nevertheless, we mainly investigate 10-day pre- and post-period since a narrow window is expected to minimize the possibility of capturing confounding effects [31].

**Table 4:** Average effect on days after celebrity death incidents.

	(1)	(2)	(3)
Window period	10 days	20 days	40 days
Average Effect (IRR)	1.193***	1.172***	1.141***
	(0.0258)	(0.0175)	(0.0128)
Observations	5,113	5,113	5,113

Note: Robust standard errors are in parentheses.

*** p<0.01, ** p<0.05, * p<0.1

**Fig 4:**
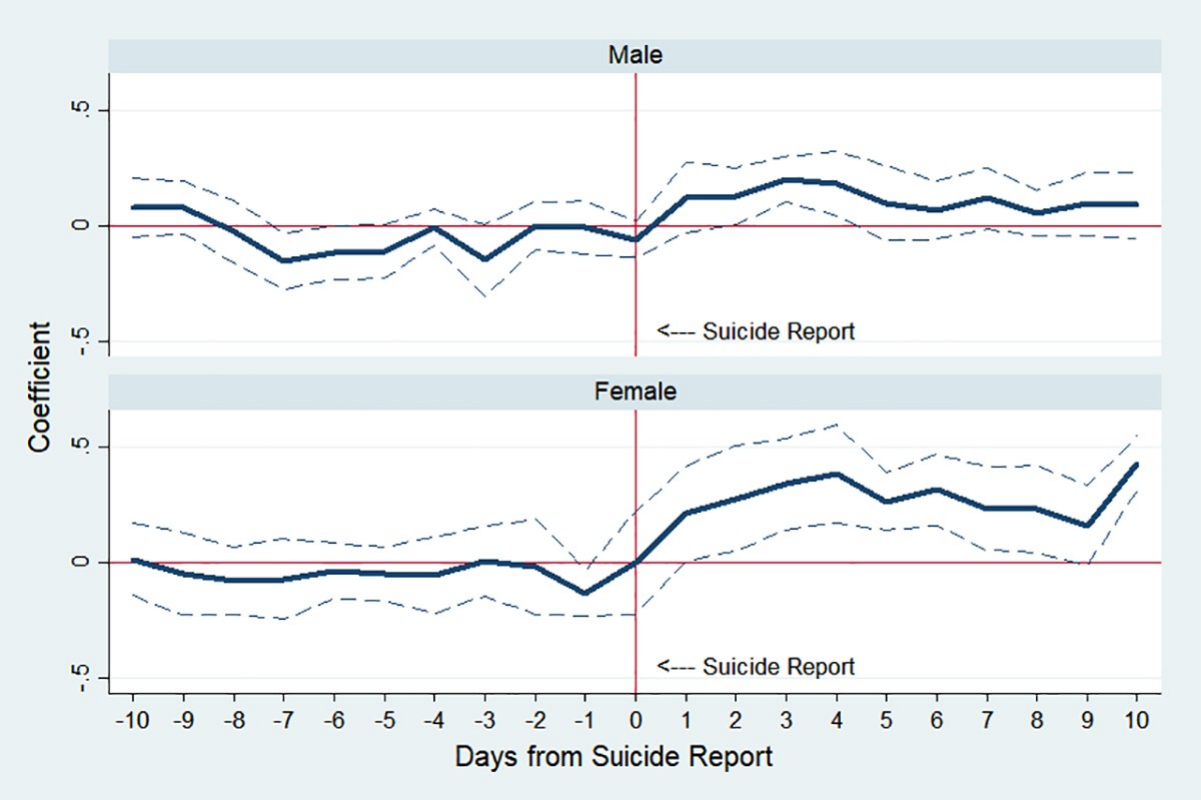
Estimated changes in total suicides by gender before and after media reports on celebrity suicide. Note: The *y*-axis indicates an approximate percent change in public suicide by corresponding day, which is the estimated *β*_*k*_ in Eq (1). The dotted lines indicate 95% confidence interval.

Fig 4 plots the estimation results of male and female subgroups. In our sample, the average number of suicides of men per day is 25.3, higher than that of women, 11.8 (Table 1), but the Werther effect is greater for women. Specifically, in columns (3) and (4) of Table 3, IRR on day 4 is 1.201 (p-value<0.01) for men, whereas that of women is 1.466 (p-value<0.001). This finding is consistent with previous studies that female and younger subgroups are more vulnerable to copycat suicides [43, 44].

**Fig 5:**
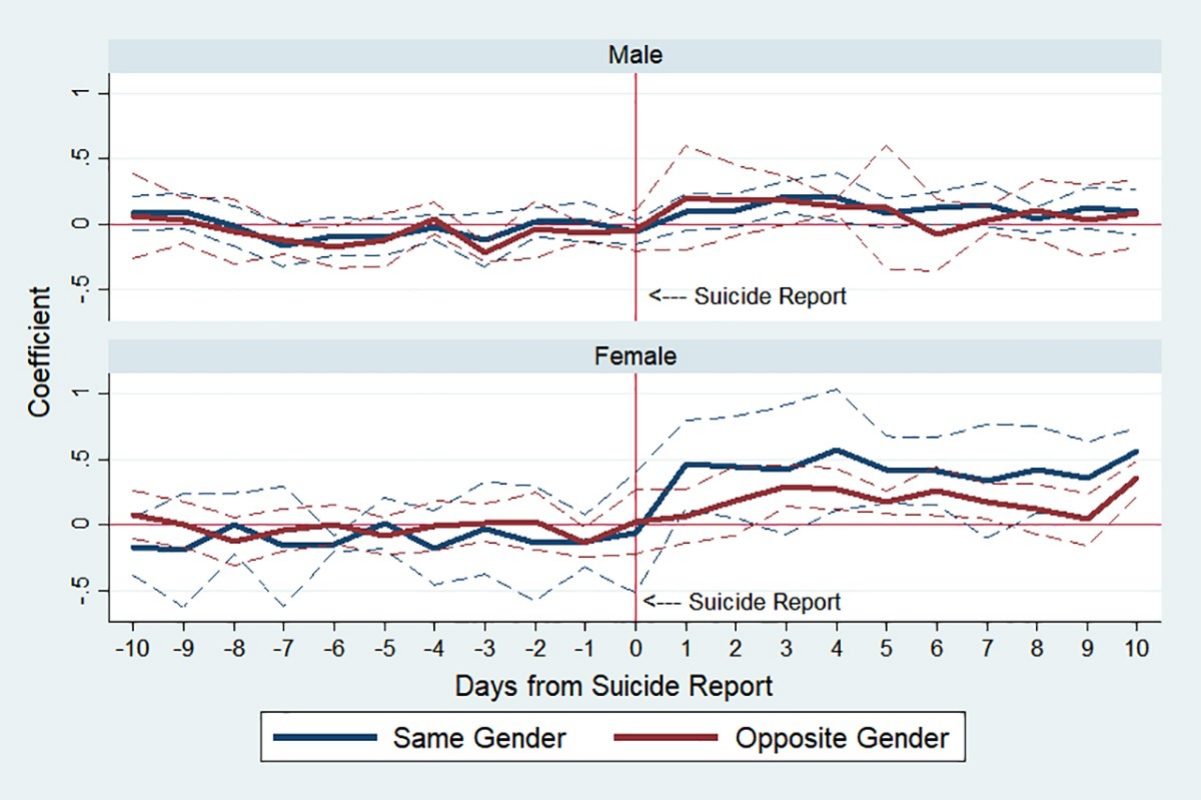
Estimated changes in total suicides by gender before and after media reports on celebrity suicide. Note: “Same Gender” refers to celebrity suicides of the same gender, while “Opposite Gender” refers to those of the opposite gender. For example, the effect of female celebrity suicides on the female sample is the blue line denoted as “Same Gender” while the effect of male celebrity suicides on the female sample is the red line denoted as “Opposite Gender.” The *y*-axis indicates an approximate percent change in public suicide by corresponding day, which is the estimated *β*_*k*_ in Eq (1). The dotted lines indicate 95% confidence interval.

Additionally, Fig 5 shows that both females and males react more strongly to suicides of the same gender celebrities. This effect is more prominently observed for the female group than for the male group as shown in the bottom panel of Fig 5. For females, the difference between the effects of celebrity suicides of the same gender and the opposite gender is notably large, while for males, the difference is not so large.

**Fig 6:**
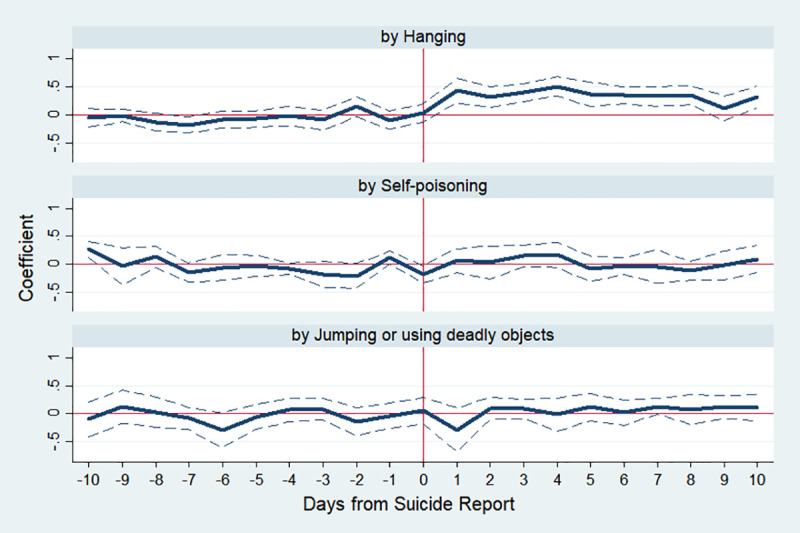
Estimated changes in total suicides by suicidal method before and after media reports on celebrity who committed suicide by hanging. Note: The *y*-axis indicates an approximate percent change in public suicide by corresponding day, which is the estimated *β*_*k*_ in Eq (1). The dotted lines indicate 95% confidence interval.

We further conduct a separate analysis by the method of suicide such as hanging, self-poisoning, jumping from a dangerous place, and using deadly objects. In our sample, the average number of people who commit suicide by hanging, self-poisoning, and jumping or using deadly objects is 18.9, 10.4 and 5.9 per day, respectively (Table 1). The analysis is based on data from nine celebrities who hanged themselves. Fig 6 shows how the number of suicides in the population changes depending on the way of suicide. The top panel of Fig 6 shows that when the celebrity suicide by hanging is reported in the media, the number of public suicides by the same method increases significantly, which is strong evidence of copycat suicide. However, as can be seen in the remaining panels of Fig 6, the number of people who commit suicide in other ways does not increase significantly. In detail, IRR on day 1 by hanging is 1.539 (p-value <0.001) in column (5) of Table 3, whereas others are not statistically significant.

We also investigate the effectiveness of “Suicide Reporting Guideline 2.0” which the Korean government implemented on September 9, 2013, to reduce the negative impact of sensational suicide reports. The guideline requests to minimize reports on suicide and refrain from using the word “suicide” as well as other sensational expressions. In addition, broad casting media including newspapers should minimize detailed information about suicide, avoid romanticization or making statements about suicide or those who commit suicide, and inform the public about the consequences of suicide. Further, they should provide accurate information about suicide prevention and not utilize the suicide report to question social issues. In all analyses so far, implementation of this policy has been used as a control variable, and the IRR estimate less than 1 of this variable means that suicide has decreased since this policy was implemented. Furthermore, we divide our sample into before and after the policy.

**Fig 7:**
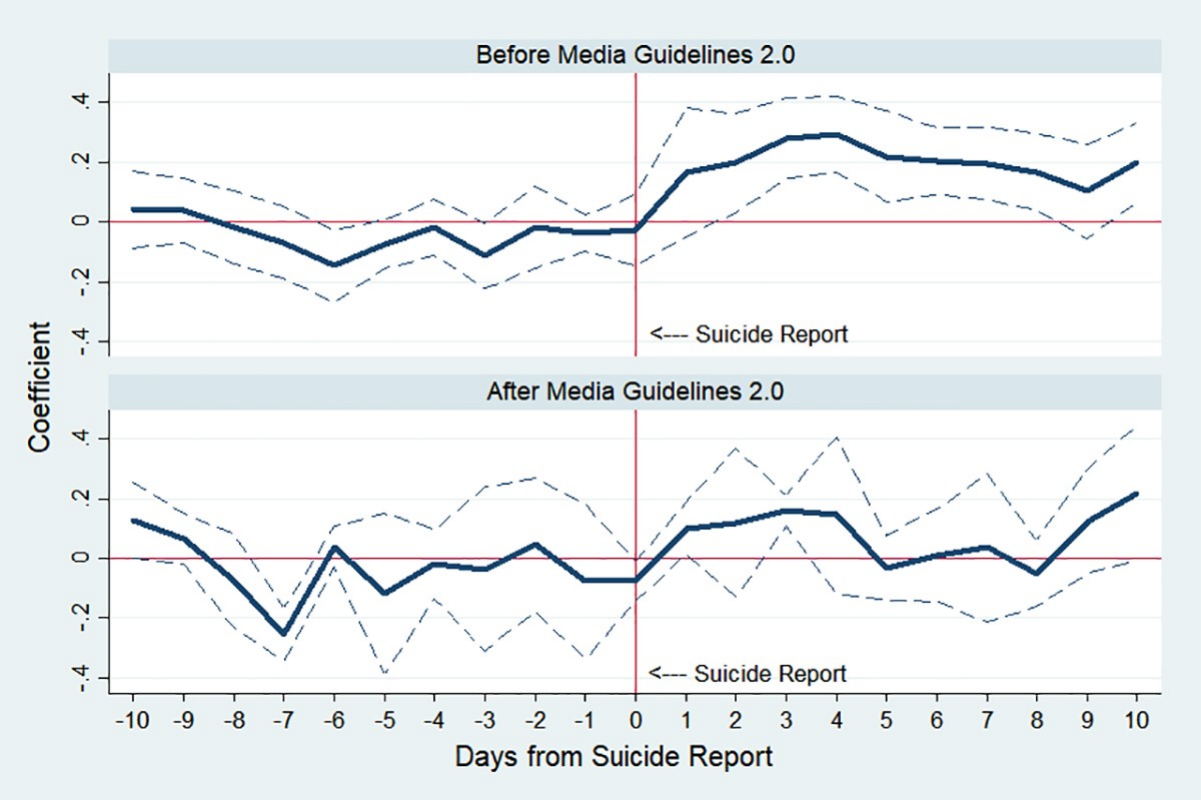
Estimated changes in total suicides before and after media reports on celebrity suicide. Note: The top panel shows the result before implementing “Suicide Reporting Guideline 2.0” and the bottom panel reports it after the implementation. The *y*-axis indicates an approximate percent change in public suicide by corresponding day, which is the estimated *β*_*k*_ in Eq (1). The dotted lines indicate 95% confidence interval.

Two graphs in Fig 7 show the influence of celebrity suicides on the public for periods before and after the implementation of the guideline. Both graphs show that the number of suicides increases after celebrity suicides, but the magnitude is smaller in the period after the release of the guideline. Admittedly, the interpretation of this finding is limited since the characteristics of celebrities who committed suicide before and after the policy change are not identical. But this is suggestive evidence that responsible and cautious media coverage of suicide could reduce its negative impact.

**Fig 8:**
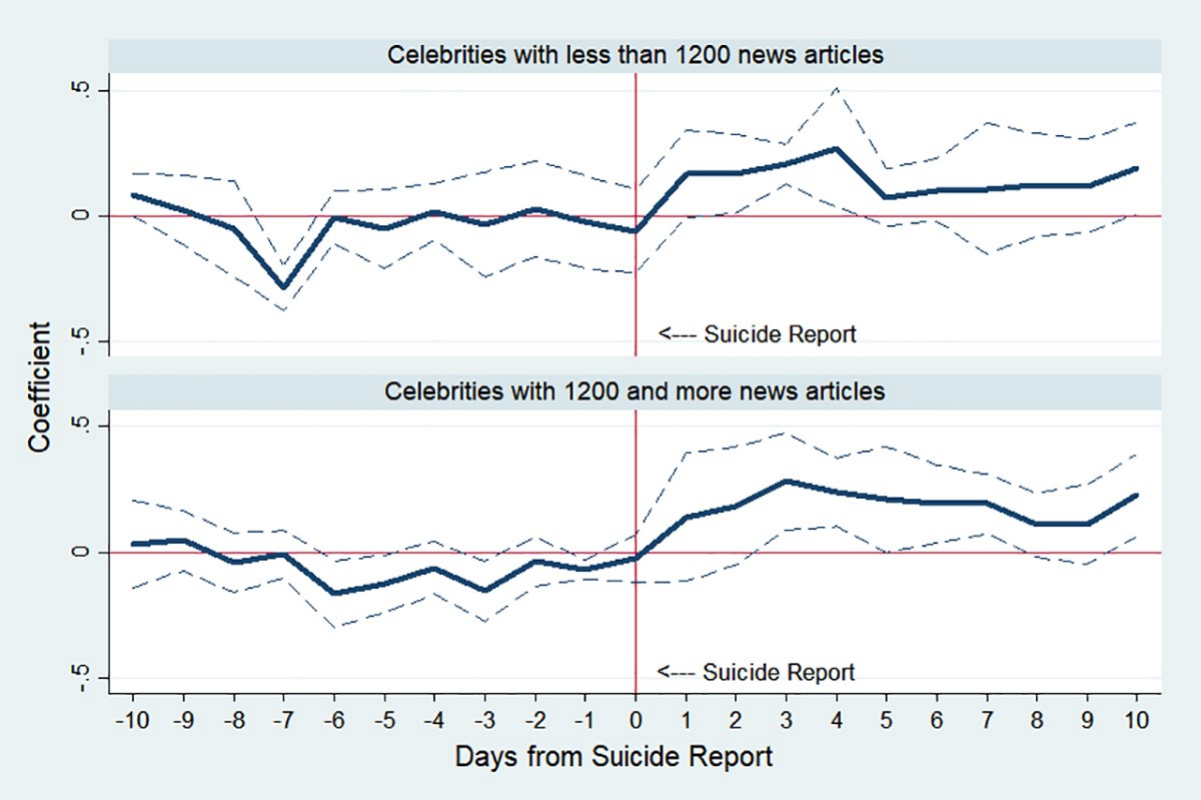
Estimated changes in total suicides before and after celebrity suicide media reports using the number of online news articles. Note: The top panel shows results using celebrity suicide reports of less than 1200 news articles and the bottom panel reports results using celebrity suicide reports with 1200 or more news articles. The number 1,200 was selected to divide the celebrities into two groups with enough and even sample sizes. There are seven cases of more than 1,200 news reports and six cases of lower than 1,200. The *y*-axis indicates an approximate percent change in public suicide by corresponding day, which is the estimated *β*_*k*_ in Eq (1). The dotted lines indicate 95% confidence interval.

Fig 8 shows how much the Werther effect varies depending on the amount of media coverage of suicide cases. We separated the celebrities according to the number of online news and classified them as highly publicized cases when the number of news articles in Table 2 is more than 1,200. The number 1,200 was selected to divide the celebrities into two groups with enough and even sample sizes. There are seven cases of more than 1,200 news reports and six cases of lower than 1,200. The two graphs in Fig 8 show that more reported suicide incidents have a relatively stronger impact than less reported cases. In Fig A3 in S1 Appendix, moreover, the same analysis with the 40-day window provides evidence that the more articles the media reported, the longer and stronger the effects last. This finding is also confirmed by comparing IRR provided in Table A1 in S1 Appendix.

## Robustness checks

**Fig 9:**
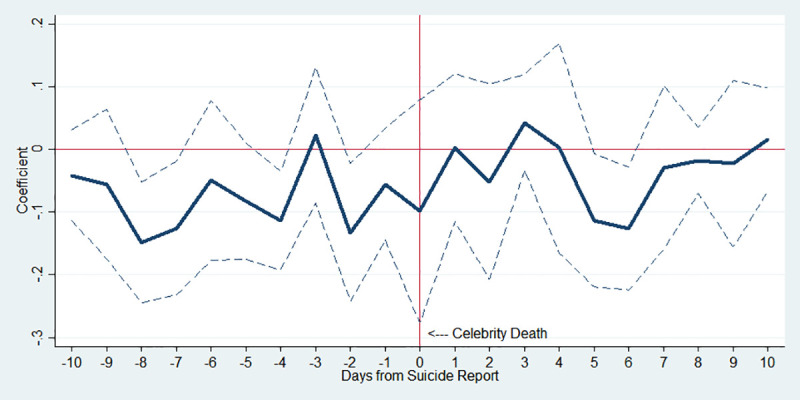
Estimated changes in total suicides before and after media reports on celebrity who died from accidents or illness, not suicide. Note: The *y*-axis indicates an approximate percent change in public suicide by corresponding day, which is the estimated *β*_*k*_ in Eq (1). The dotted lines indicate 95% confidence interval.

Meanwhile, it must be distinguished whether the increase in the number of suicides is a result of copycat suicides or simply caused by negative emotion due to the death of an enviable celebrity. Therefore, we also analyze the impact of celebrity deaths instead of celebrity suicides. The list of celebrities who died from an accident or illness can be found in Table 2, and the estimation result is shown in Fig 9 and column (6) of Table 3. As expected, unless the cause of celebrity deaths is suicide, there is no effect on suicides of the public. This result, which shows that people only respond to celebrity suicide reports, supports claims that indiscriminate media reports on celebrity suicides trigger imitative suicides.

**Fig 10:**
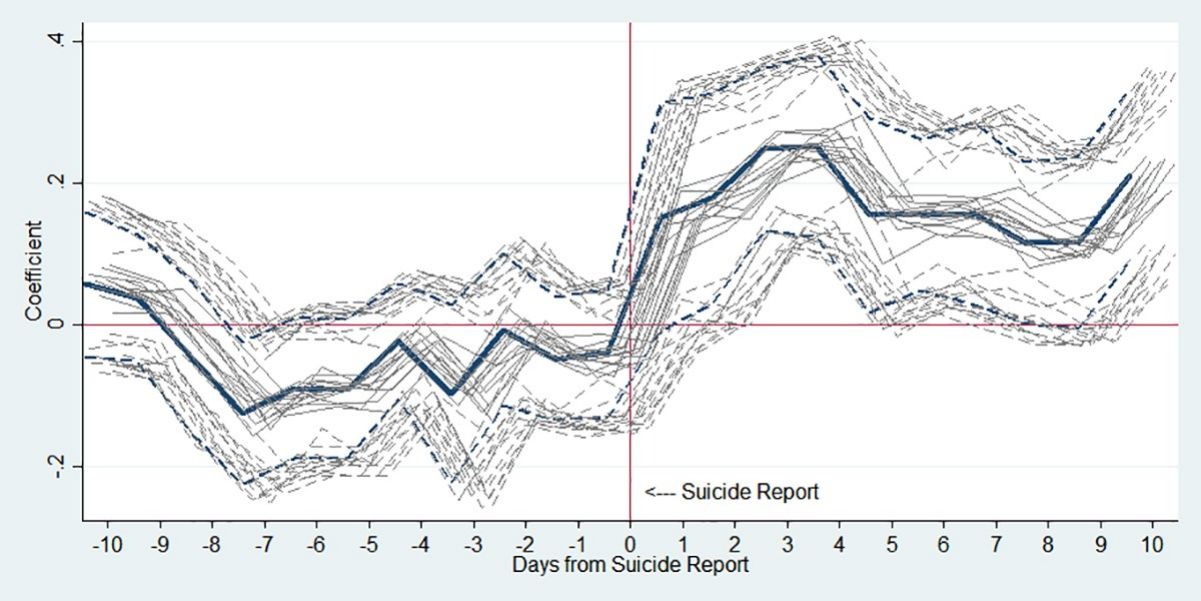
Estimated changes in total suicides before and after media reports on celebrity suicide. Note: The thick blue line is the main result of the top panel of Fig 2 using all 13 celebrity suicides. The light gray lines each represent results from 12 celebrity suicides after omitting each of the 13 celebrity suicides. The *y*-axis indicates an approximate percent change in public suicide by corresponding day, which is the estimated *β*_*k*_ in Eq (1). The dotted lines indicate 95% confidence interval.

It is also a plausible doubt that the effects of one or two of the 13 highly publicized suicides are so dominant that the results are misinterpreted. Therefore, we conduct the analysis using only 12 cases out of 13 celebrities for the robustness check, and the results are shown in Fig 10. As shown in the figure, results from 12 celebrity suicides after omitting each of the 13 celebrity suicides are similar to the main result in Fig 2.

Two studies have focused on two celebrities, Choi and Roh, whose suicides are the most well-known cases in Korea and found strong influences of these particular suicides [15, 16]. Since it is possible that these two suicide cases may drive our results, we conduct an analysis with only 11 celebrities excluding Choi and Roh. The result is similar to the main result in Fig 2, although the significance level slightly decreases because two notable events are excluded.

**Fig 11:**
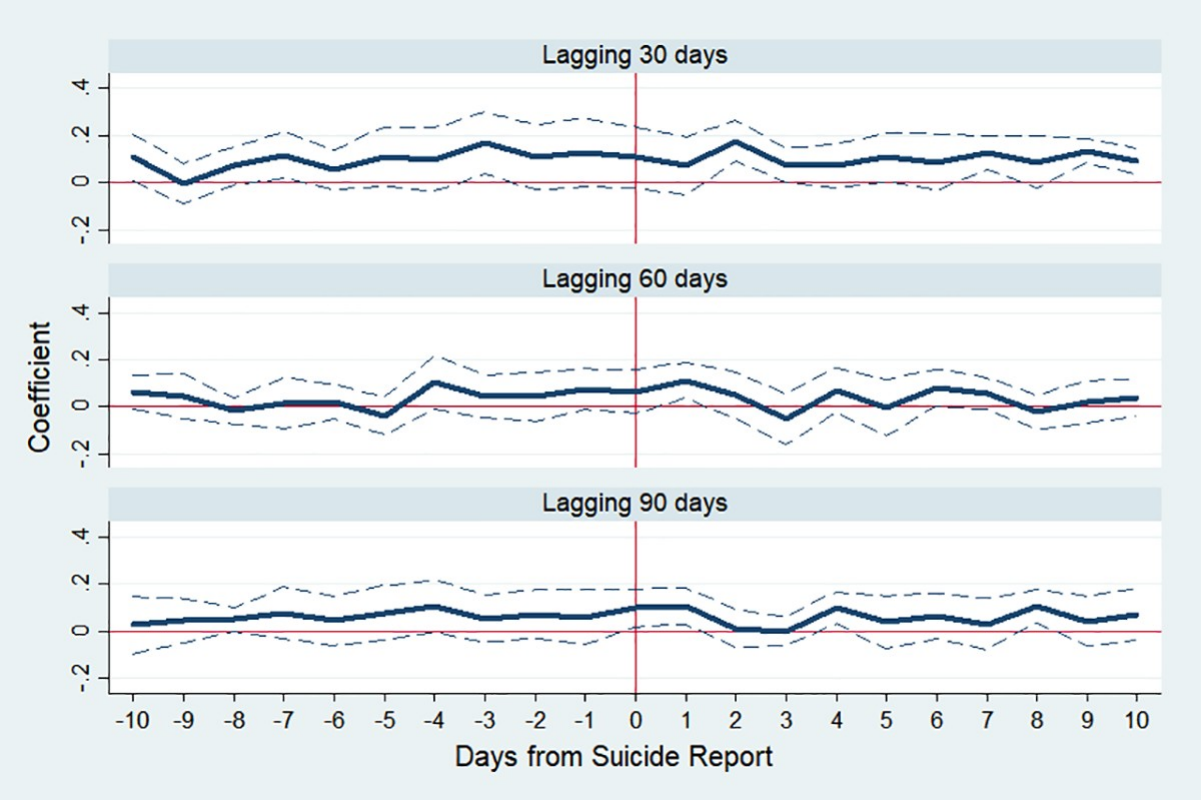
Estimated changes in total suicides using different lags. Note: We assign lags of 30, 60, and 90 days to the actual dates of celebrity suicides. The *y*-axis indicates an approximate percent change in public suicide by corresponding day, which is the estimated *β*_*k*_ in Eq (1). The dotted lines indicate 95% confidence interval.

Moreover, we conduct a placebo test by assigning the timing of celebrity suicides differently from the actual dates. Fig 11 shows that there is no significant impact of the suicides when we set the incident dates after 30, 60, and 90 days from the actual dates.

## Discussion

Using daily suicide data, this paper empirically examines whether there are copycat suicides after reports on celebrity suicides in Korea. The findings from the Poisson regression model help us understand the specific timing and duration of the Werther effect. Specifically, it is observed that public suicides are likely to follow the suicide of a celebrity, and the effect is most powerful in three to four days after the suicide report. Furthermore, the results show that female and younger subgroups commit more suicides after highly-publicized suicides. It is also found that the public reacts more strongly to suicide incidents of celebrities of the same gender and even imitates the methods of suicide used by celebrities. Moreover, although there is a limitation in interpretation, suggestive evidence shows that the media guidelines of the government seem to be effective in reducing copycat suicides. On the other hand, celebrity deaths by accidents or diseases have no effect on the public suicide. Although these findings are useful for understanding suicide risk factors, the results do not rule out the role of other factors in influencing suicide risk; copycat suicide explanation could co-exist with other conventional explanations.

The findings call for more attention to the potential unintended consequences of celebrity suicides and the possible policy remedies to safeguard the public against depression and suicide risk. In particular, policymakers can minimize the suicide risk associated with highly publicized suicide stories as more frequent and indiscriminate media reports are likely to have a negative impact on the public. As suicide is often related to mental illness, conducting mental health assessments and providing services after celebrity suicides can also potentially reduce the suicide risk.

Moreover, ever since classrooms have moved online due to the COVID-19 pandemic, students have been more likely to be exposed to new types of media reports than ever. As shown in our paper, they also belong to the age group most vulnerable to stimulating online news such as celebrity suicides. The new media such as social media, single-person media, and other Internet-based media provides them with a lot of unfiltered and sensational information and is usually associated negatively with the mental health of teenagers [45, 46]. Therefore, it is crucial to provide regulations not only for traditional media but also for these new platforms.

## Supporting information

S1 Appendix (DOCX)

S1 Data (DTA)

## References

[1] Eckersley R, Dear K. Cultural correlates of youth suicide. Social Science & Medicine. 2002;55(11):1891–904. 10.1016/s0277-9536(01)00319-7 12406459

[2] Wasserman D, Cheng Q, Jiang G-X. Global suicide rates among young people aged 15–19. World Psychiatry. 2005; 4(2):114–120 16633527 PMC1414751

[3] Shin H-Y, Kim J, Lee S, Park MS, Park S, Huh S. Cause-of-death statistics in 2018 in the Republic of Korea. Journal of the Korean Medical Association. 2020;63(5):286–97.

[4] Gould MS, Petrie K, Kleinman MH, Wallenstein S. Clustering of Attempted Suicide: New Zealand National Data. International Journal of Epidemiology. 1994;23(6):1185–9. 10.1093/ije/23.6.1185 7721521

[5] Cutler DM, Glaeser EL, Norberg KE. Explaining the rise in youth suicide. In: Gruber J, editor. Risky behavior among youths: An economic analysis. Chicago: University of Chicago Press. 2001; pp. 219–269.

[6] Bearman PS, Moody J. Suicide and Friendships Among American Adolescents. American Journal of Public Health. 2004;94(1):89–95. 10.2105/ajph.94.1.89 14713704 PMC1449832

[7] Abrutyn S, Mueller AS. Are Suicidal Behaviors Contagious in Adolescence? Using Longitudinal Data to Examine Suicide Suggestion. American Sociological Review. 2014;79(2):211–27. 10.1177/0003122413519445 26069341 PMC4461080

[8] Mueller AS, Abrutyn S. Suicidal Disclosures among Friends. Journal of Health and Social Behavior. 2015;56(1):131–48. 10.1177/0022146514568793 25722129 PMC4472458

[9] Eisenberg D, Golberstein E, Whitlock JL, Downs MF. Social Contagion Of Mental Health: Evidence From College Roommates. Health Economics. 2012;22(8):965–86. 10.1002/hec.2873 23055446 PMC4381550

[10] Eisenberg D, Golberstein E, Whitlock JL. Peer effects on risky behaviors: New evidence from college roommate assignments. Journal of Health Economics. 2014;33:126–38. 10.1016/j.jhealeco.2013.11.006 24316458

[11] Card D, Giuliano L. Peer Effects and Multiple Equilibria in the Risky Behavior of Friends. Review of Economics and Statistics. 2013;95(4):1130–49.

[12] Chen J, Choi YJ, Mori K, Sawada Y, Sugano S. Those who are left behind: an estimate of the number of family members of suicide victims in Japan. Social Indicators Research. 2009;94(3):535–44.

[13] Durkheim E. Suicide, a study in sociology [1897]. Translated by JA Spaulding and G. Simpson. Glencoe, Illinois: The Free Press; 1951.

[14] Wray M, Colen C, Pescosolido B. The sociology of suicide. Annual Review of Sociology. 2011;37.

[15] Kwon CH, Lee JH, Yun YH. The Revealed Influence of Celebrities’ Suicide on an Attempted Suicide of the Public. Journal of The Korean Society of Health Informatics and Statistics. 2012;37(1):22–30.

[16] Kim J-H, Park E-C, Nam J-M, Park S, Cho J, Kim S-J, et al. The Werther Effect of Two Celebrity Suicides: an Entertainer and a Politician. PLoS ONE. 2013;8(12): e84876. 10.1371/journal.pone.0084876 24386428 PMC3873447

[17] Jang SA, Sung JM, Park JY, Jeon WT. Copycat Suicide Induced by Entertainment Celebrity Suicides in South Korea. Psychiatry Investigation. 2016;13(1):74–81. 10.4306/pi.2016.13.1.74 26766949 PMC4701688

[18] Phillips DP. The Influence of Suggestion on Suicide: Substantive and Theoretical Implications of the Werther Effect. American Sociological Review. 1974;39(3):340–54. 11630757

[19] Wasserman IM. Imitation and Suicide: A Reexamination of the Werther Effect. American Sociological Review. 1984;49(3):427–36.

[20] Stack S. A reanalysis of the impact of non celebrity suicides. Social Psychiatry and Psychiatric Epidemiology. 1990;25(5):269–73. 10.1007/BF00788648 2237608

[21] Stack S. Celebrities and Suicide: A Taxonomy and Analysis, 1948–1983. American Sociological Review. 1987;52(3):401–12. 11613886

[22] Bollen KA, Phillips DP. Imitative Suicides: A National Study of the Effects of Television News Stories. American Sociological Review. 1982;47(6):802–9. 11630893

[23] Cheng ATA, Hawton K, Lee CTC, Chen THH. The influence of media reporting of the suicide of a celebrity on suicide rates: a population-based study. International Journal of Epidemiology. 2007;36(6):1229–34. 10.1093/ije/dym196 17905808

[24] Ladwig K-H, Kunrath S, Lukaschek K, Baumert J. The railway suicide death of a famous German football player: Impact on the subsequent frequency of railway suicide acts in Germany. Journal of Affective Disorders. 2012;136(1–2):194–8. 10.1016/j.jad.2011.09.044 22036798

[25] Hegerl U, Koburger N, Rummel-Kluge C, Gravert C, Walden M, Mergl R. One followed by many?—Long-term effects of a celebrity suicide on the number of suicidal acts on the German railway net. Journal of Affective Disorders. 2013;146(1):39–44. 10.1016/j.jad.2012.08.032 23040873

[26] Ueda M, Mori K, Matsubayashi T. The effects of media reports of suicides by well-known figures between 1989 and 2010 in Japan. International Journal of Epidemiology. 2014;43(2):623–9. 10.1093/ije/dyu056 24639437

[27] Ueda M, Mori K, Matsubayashi T, Sawada Y. Tweeting celebrity suicides: Users’ reaction to prominent suicide deaths on Twitter and subsequent increases in actual suicides. Social Science & Medicine. 2017;189:158–66. 10.1016/j.socscimed.2017.06.032 28705550

[28] Bridge JA, Greenhouse JB, Ruch D, Stevens J, Ackerman J, Sheftall AH, et al. Association Between the Release of Netflix’s 13 Reasons Why and Suicide Rates in the United States: An Interrupted Time Series Analysis. Journal of the American Academy of Child & Adolescent Psychiatry. 2020;59(2):236–43. 10.1016/j.jaac.2019.04.020 31042568 PMC6817407

[29] Fu K-W, Chan CH. A Study of the Impact of Thirteen Celebrity Suicides on Subsequent Suicide Rates in South Korea from 2005 to 2009. PLoS ONE. 2013;8(1): e53870. 10.1371/journal.pone.0053870 23342026 PMC3547049

[30] Jonas K. Modelling and suicide: a test of the Werther effect. British Journal of Social Psychology. 1992;31(4):295–306. 10.1111/j.2044-8309.1992.tb00974.x 1472984

[31] Hong SC, Lee J. People on the verge of death: evidence from impacts of celebrity suicides. Applied Economics. 2015;47(7):710–24.

[32] Rubin R, McHugh M. Development of parasocial interaction relationships. Journal of Broadcasting and Electronic Media. 1987;31:279–92.

[33] Moksony F, Hegedűs R. The use of Poisson regression in the sociological study of suicide. Corvinus Journal of Sociology and Social Policy. 2015;5(2).

[34] Deisenhammer EA. Weather and suicide: the present state of knowledge on the association of meteorological factors with suicidal behaviour. Acta Psychiatrica Scandinavica. 2003;108(6):402–9. 10.1046/j.0001-690x.2003.00209.x 14616220

[35] Lee HC, Lin HC, Tsai SY, Li CY, Chen CC, Huang CC. Suicide rates and the association with climate: a population-based study. Journal of affective disorders. 2006;92(2–3):221–6. 10.1016/j.jad.2006.01.026 16513180

[36] Lin C-L, Liu T-C, Chen C-S. The association between attempted suicide and stock price movements: evidence from Taiwan. Psychiatry research. 2017;254:323–31. 10.1016/j.psychres.2017.05.004 28505601

[37] Breuer C. Unemployment and suicide mortality: evidence from regional panel data in Europe. Health economics. 2015;24(8):936–50. 10.1002/hec.3073 24934277

[38] Zhang J, Ma J, Jia C, Sun J, Guo X, Xu A, et al. Economic growth and suicide rate changes: a case in China from 1982 to 2005. European psychiatry. 2010;25(3):159–63. 10.1016/j.eurpsy.2009.07.013 19926256

[39] Stack S. The impact of divorce on suicide in Norway. Journal of Marriage and the Family. 1989:229–38.

[40] Stack S. The effect of divorce on suicide in Denmark. Sociological Quarterly. 1990;31(3):359–70.

[41] Korean Ministry of Health and Welfare. Suicide reporting guideline 2.0 (in Korean) [Internet]. [cited on 17 November 2020]. Available from: http://www.korea.kr/archive/expDocView.do?docId=34401

[42] Gould MS, Greenberg TED, Velting DM, Shaffer D. Youth suicide risk and preventive interventions: a review of the past 10 years. Journal of the American Academy of Child & Adolescent Psychiatry. 2003;42(4):386–405. 10.1097/01.CHI.0000046821.95464.CF 12649626

[43] Yi H, Hwang J, Bae H-J, Kim N. Age and sex subgroups vulnerable to copycat suicide: evaluation of nationwide data in South Korea. Scientific Reports. 2019;9(1):1–9. 10.1038/s41598-018-37186-2 31754190 PMC6872728

[44] Myung W, Won H-H, Fava M, Mischoulon D, Yeung A, Lee D, et al. Celebrity Suicides and Their Differential Influence on Suicides in the General Population: A National Population-Based Study in Korea. Psychiatry Investigation. 2015;12(2):204–11. 10.4306/pi.2015.12.2.204 25866521 PMC4390591

[45] Twenge JM, Joiner TE, Rogers ML, Martin GN. Increases in Depressive Symptoms, Suicide-Related Outcomes, and Suicide Rates Among U.S. Adolescents After 2010 and Links to Increased New Media Screen Time. Clinical Psychological Science. 2017;6(1):3–17.

[46] Bányai F, Zsila Á, Király O, Maraz A, Elekes Z, Griffiths MD, et al. Problematic Social Media Use: Results from a Large-Scale Nationally Representative Adolescent Sample. Plos One. 2017;12(1): e0169839. 10.1371/journal.pone.0169839 28068404 PMC5222338

