# Supplementary material for: The Werther effect of celebrity suicides: Evidence from South Korea
Source: PLoS One. 2021 Apr 28;16(4):e0249896. doi: 10.1371/journal.pone.0249896 (PMC8081220; doi:10.1371/journal.pone.0249896)
Supplement: S1 Appendix — (DOCX) [file pone.0249896.s001.docx]

**S1 Appendix**


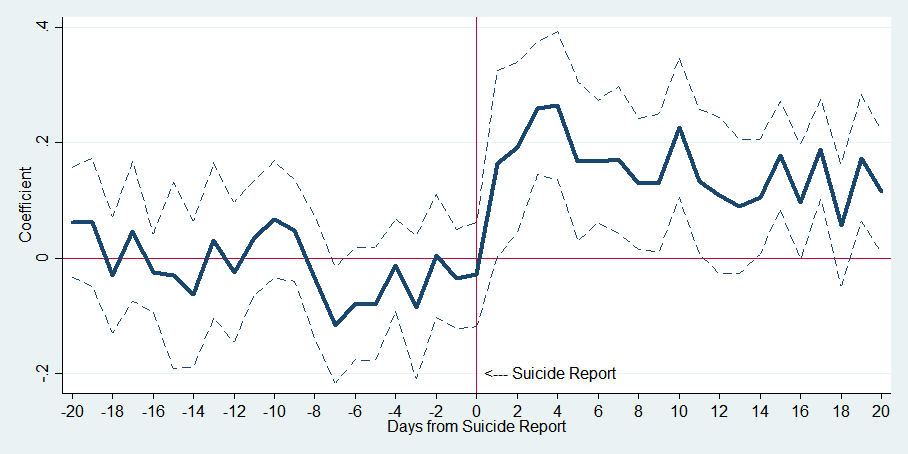


**Figure A1. Estimated changes in total suicides before and after celebrity suicide media reports using a 20 pre- and post-period.**

Note: The $y$-axis indicates an approximate percent change in public suicide by corresponding day, which is the estimated $\beta_{k}$ in equation (1). The dotted lines indicate 95% confidence interval.


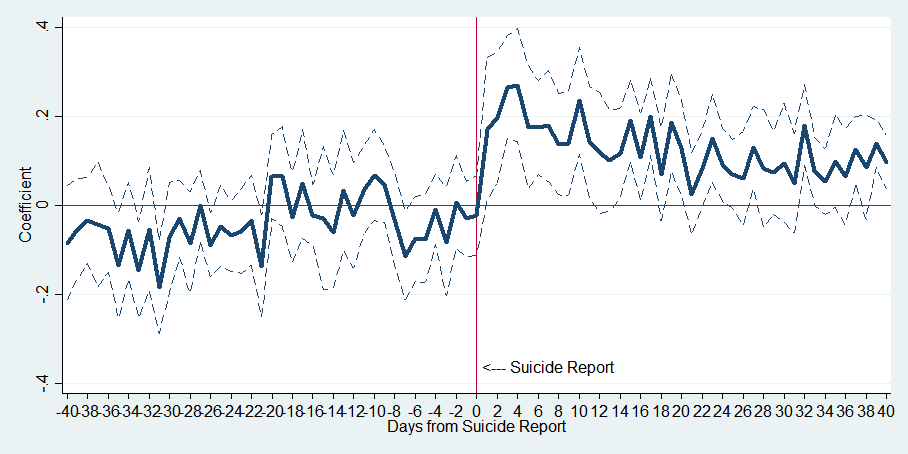


**Figure A2. Estimated changes in total suicides before and after celebrity suicide media reports using a 40 pre- and post-period.**

Note: The $y$-axis indicates an approximate percent change in public suicide by corresponding day, which is the estimated $\beta_{k}$ in equation (1). The dotted lines indicate 95% confidence interval.


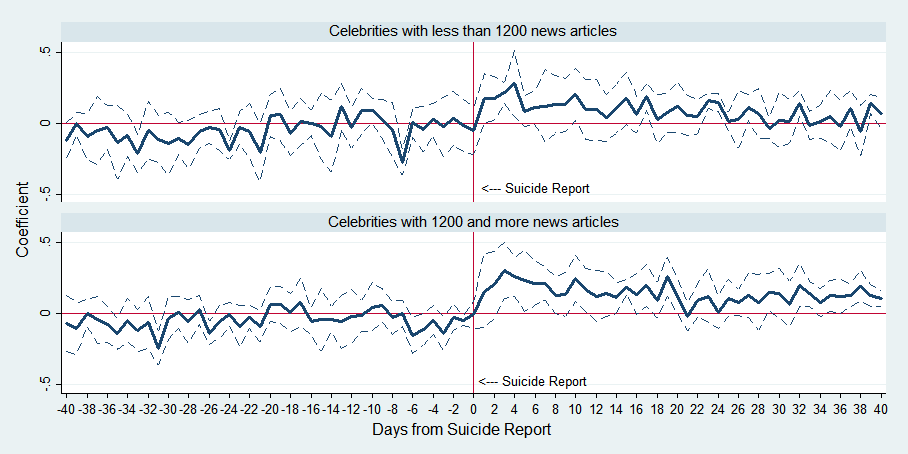


**Figure A3. Estimated changes in total suicides before and after celebrity suicide media reports according to the number of online news articles (40 pre- and post- period).**

Note: The top panel shows results using celebrity suicide reports of less than 1,200 news articles and the bottom panel reports results using celebrity suicide reports with 1,200 or more news articles. The $y$-axis indicates an approximate percent change in public suicide by corresponding day, which is the estimated $\beta_{k}$ in equation (1). The dotted lines indicate 95% confidence interval.

Table A1. Average effect on days after celebrity death incidents by the number of online news

|  | (1) | (2) |
| --- | --- | --- |
|  | Less reported | More reported |
| Average Effect (1-10 days) | 1.179*** | 1.238*** |
|  | (0.0360) | (0.0379) |
| Average Effect (11-20 days) | 1.103*** | 1.170*** |
|  | (0.0318) | (0.0289) |
| Average Effect (21-40 days) | 1.056*** | 1.119*** |
|  | (0.0184) | (0.0196) |
| Constant | 35.23*** | 35.45*** |
|  | (2.831) | (2.917) |
| Observations | 5,113 | 5,113 |

Note: Column (1) shows estimates using celebrity suicide reports of less than 1,200 news articles and column (2) shows estimates using celebrity suicide reports with 1,200 or more news articles. Robust standard errors are in parentheses. *** p<0.01, ** p<0.05, * p<0.1
